# Impacts of COVID-19 on HIV/AIDS-Related Services in California

**DOI:** 10.1177/23259582221128500

**Published:** 2022-10-09

**Authors:** Kimberly A. Koester, Shannon M. Fuller, Wayne T. Steward, Emily A. Arnold

**Affiliations:** 1 Division of Prevention Science, University of California San Francisco, San Francisco, CA, USA

**Keywords:** COVID-19, HIV/AIDS, rapid assessment, qualitative methods

## Abstract

The degree to which COVID-19 has disrupted the advances in reducing new HIV infections and preventing AIDS-related deaths is unknown. We present findings related to the effect COVID-19 had on HIV, sexual health and harm reduction service delivery in the state of California. We conducted a qualitative rapid assessment with health care providers, as well as representatives from non-medical support service agencies serving clients living with HIV in a range of counties in California. Some organizations adapted fairly easily while others struggled or were unable to adapt at all. Clinics were better positioned than community-based organizations to accommodate COVID restrictions and to quickly reestablish services. Influential forces that softened or calcified the hardships created by COVID-19 included influx of funding, flexibility in managing funds, networking and relationships, and workforce vulnerabilities. These data clearly suggest that an enhanced level of flexibility within funding streams and reporting requirements should be continued**.**

## Introduction

Approximately 30,635 individuals living in the United States and its six territories were newly diagnosed with HIV in 2020.^1^ This figure represents a 17% decline in new HIV infections since 2019.^1^ The Centers for Disease Control and Prevention (CDC) emphasized using caution when interpreting the data due to the impact of the COVID-19 pandemic on access to HIV testing. That said, initiatives to “End the HIV Epidemic” have led to widescale efforts to reduce new infections in key areas of the US and while the country is far from achieving the national strategic plan to end HIV by 2030, steady progress has been made.^2^ For example, California has set goals to increase linkage and viral suppression rates to 95% by 2025. As of 2020, 71.8% were in care and 63% had achieved viral suppression.^3^

The degree to which COVID-19 has disrupted the advances in reducing new HIV infections and preventing AIDS-related deaths will take time to fully determine and may differ dramatically across the globe. A modeling study on HIV transmission rates conducted in 32 cities in the US projected an initial decline in new HIV diagnoses in 2020 followed by an increase in 2021 or 2022.^4^ The investigators modeled both liberal and conservative scenarios about pandemic impact and produced corresponding estimates ranging from 8% lower HIV incidence to 11% higher incidence from 2020–2025. These projections highlight the uncertainty of COVID-19’s impact on HIV transmission, including the possibility of delayed detection of new cases of HIV. The authors emphasized the importance of maintaining continuity of HIV care and prevention resources as much as possible to minimize the damage of care disruptions.^4^ Elsewhere, the World Health Organization reported significant disruptions in HIV-, Hepatitis-, and sexually transmitted infection (STI)- related services in low resource settings, with 36 countries reporting disrupted access to antiretroviral therapy (ART) and 73 countries at risk of an ART supply disruption or stock out.^5^

With this article, we aim to build on the accumulating evidence base about the disruptive impacts COVID-19 has had on HIV service delivery. We present findings related to the effect COVID-19 had on HIV, sexual health and harm reduction service delivery, including care, treatment, and prevention, in the state of California.

## Methods

### Study Design, Sampling & Recruitment

The California HIV Policy Research Centers, based in the northern and southern parts of the state, conducted a qualitative rapid assessment^6^ with health care providers, as well as with leaders and representatives from non-medical support service agencies serving clients living with HIV. Early in the pandemic, the centers administered a survey to HIV clinics and service agencies across California to understand how their organizations were being affected by the COVID-19 pandemic.^7^ We used findings from the survey to identify topics for exploration in qualitative interviews. Survey participants had also provided contact information and indicated a willingness to be contacted for further research, providing a means of recruiting potential informants. Survey responses were reviewed and discussed as a team, and a subset of survey respondents were recruited via email for an interview. Of particular relevance, all survey respondents had been asked to identify up to three areas of greatest need, providing a sense for the diversity and range of challenges being faced by different organizations. These data allowed the investigative team to identify a set of potential agencies facing different types of challenges. Outreach was conducted iteratively as we completed interviews, following theoretical sampling principles.^8^ In addition to recruiting prior survey respondents, we also contacted representatives from HIV organizations in the state using publicly available clinic/agency contact information posted on websites. This helped to ensure the sample reflected a mix of different agency types and areas of the state. Representatives from a total of 18 agencies were approached and 15 agencies agreed to participate. All participants were interviewed individually, except for one agency that requested having two representatives who could speak to different aspects of clinical service and administration.

Each individual who participated in an interview received a $75 gift card if their workplace permitted them to receive the incentive. All study procedures were reviewed and declared exempt by the Institutional Review Board at University of California, San Francisco (UCSF) (IRB protocol #:20-31486). All participants provided verbal consent.

### Data Collection

Between October 2020 and February 2021, authors EA and SF conducted semi-structured interviews via videoconference or audio-only depending on the participant’s preference. The interviews lasted approximately 60 minutes, were audio recorded, and professionally transcribed. Participants received an information sheet describing the study by email in advance of the interview and provided verbal informed consent at the beginning of the call. Interview domains covered COVID-19 impacts on service delivery, HIV care and prevention, and lessons learned. A brief demographic questionnaire was administered following the interview.

### Analysis

Our analytic approach was guided by the template analysis method developed for qualitative health services research.^9^ Template analysis is a deductive form of analysis that works well for rapid qualitative analysis when the domains of interest are specified a priori. Per the steps of template analysis, summary templates were prepared for each interview transcript. The template domains followed the structure of the interviews and included the following sections: Organization Services Offered, COVID Impact on Service Delivery, Challenges for Clients/Patients, Challenges for Organization/Agency, COVID Impact on Prevention/Care Continuum, and Recommendations/Lessons Learned/Facilitators. Each section was populated with a bulleted summary of information extracted from the transcript. Once a few transcripts were summarized into templates, the main analyst (KK) began noting areas of similarities and differences across cases. Template summaries were further condensed into an analytic table to facilitate cross-case and within-case comparisons. We utilized the table to identify the most noteworthy impacts in terms of patterns and redundancy, as well as examined instances when no pattern could be discerned. We routinely returned to the full transcripts to ensure we understood the context in which the narratives on impact arose.

## Findings

We interviewed a total of 16 key informants from 15 organizations across 11 California counties. In brief, we spoke to 5 clinic-based facilities and 10 community-based organizations (CBOs) focused on social service delivery. Our analysis focuses on explaining the impacts of COVID-19 and is structured according to the organizational setting, either clinic or support services. We examined the differences in and consequences of the presence or absence of the capacity of organizations to adapt to the public health restrictions across sites. On the extremes, some adapted fairly easily while others struggled or were unable to adapt at all. A clear pattern surfaced indicating that clinics were better positioned than CBOs to accommodate COVID restrictions and to quickly reestablish services. We outline the influential forces that softened the hardships created by COVID-19.

### Impact of COVID-19 on Service Delivery

#### Clinical Sites Swiftly Pivoted to Telehealth

For the most part, interviewees across the five clinical settings portrayed their organizations as highly resilient in the face of COVID. Most were able to continue providing medical, mental health, and other supportive services to patients by shifting from in-person to telehealth delivery without a temporary cessation of services. The sole exception occurred in a health center that had its transition to telehealth delayed because they did not have sufficient numbers of clinicians with sufficient knowledge of and experience with telehealth care delivery.

Delivering care via telehealth was by far the most important shift clinics needed to make to ensure continuity of services. To put the shift in context, we sought to understand the systems of care delivery prior to the COVID-19 pandemic. None of the clinics had prior experience offering telehealth services. Therefore, they had no experience scheduling patients for telehealth visits, billing for these visits, or conducting a patient visit through videoconferencing or over the phone. One key informant explained that their lack of experience with telehealth was due to the fact that up until the COVID crisis, use of telehealth was not allowed or “not in our language” because it was not a billable expense. There was no incentive to offer telehealth visits. Despite the lack of experience with telehealth, a couple of sites described the transition to telehealth as “really quick.” In one instance, a clinic ramped up telehealth visits over the course of two weeks. In another, it took slightly longer as explained by an interviewee:

Before COVID, … about 95% of our services were face-to-face. And whether it's medical care or social services. . . We didn't do any telehealth. That was not in our language. It wasn't allowed in any of our contracts. So, it's been quite interesting to shift now, with the start of the pandemic last March, when it's about maybe 90% of the services being done through telehealth or telephone, versus in-person. - Clinic PT16

Once the Department of Health and Human Services changed policies permitting telehealth visits to be an allowable service during COVID-19, one key hurdle was cleared.^10^

In addition to having a pathway to bill for telehealth through contractual agreements, other elements were critical to fully transition to telehealth visits. These included: identifying educational resources for clinicians and patients to make optimal use of telehealth, providing resources such as updated laptop computers, headsets and web cameras, developing billing systems, and ensuring Health Insurance Portability and Accountability Act (HIPAA) compliance.

### Unique Challenges for CBOs

Our sample included 10 community-based organizations serving people living with and vulnerable to HIV, viral hepatitis, STIs, and drug overdose. Unlike the clinical sites, there were few similarities in how CBOs adjusted to the disruptions of COVID-19. Some made the transition to offering virtual services, others temporarily closed, and still others changed the types of services they offered.

When possible, CBOs restructured themselves around the needs and abilities of the clients they served. For example, some organizations chose to not offer *any* virtual programming because it did not make sense for their client base. In these cases, clients were accustomed to accessing services in-person and likely had no means to remotely interact with organizations. This was the situation for a CBO that provided legal counsel to individuals facing immigration issues, for example. Rather than offering virtual appointments, they created an in-person, appointment-only program to accommodate clients who historically would drop-in for services.

The heterogeneity in COVID-19 adaptations was, in part, influenced by whether or not the organization received federal funding from the Ryan White HIV/AIDS Program (henceforth RW funding), a payer of last resort for people living with HIV. The impact of COVID-19 on service delivery among CBOs that were not receiving RW funding was markedly distinct from those that had RW funding in place. Several of the non-RW funded organizations closed their doors for the first 2–3 months while they worked out plans to cope with the uncertainty wrought by the pandemic.

In another case, a non-RW funded CBO shifted from offering HIV and STI testing and STI treatment to addressing basic needs for people experiencing homelessness.

CBOs that shifted services from in-person to exclusively or mainly remote did so with difficultly. The challenges for some organizations included not having existing technology ie, no laptops, to support their staff to work from home. Others may have had the technology, but their client-facing staff did not have sufficient experience with technology or, in some cases, they did not have a clear idea about how to virtually carry out their work with clients thus making a rocky transition to remote work. In most cases, the organizations operating outside of the RW funding sphere had little to no influx of funds to address these technology gaps. Despite the immense challenges, we noted self-reliance among these CBOs. They operated on the beneficence of volunteers or dedicated executive directors. As one informant explained, when COVID hit the thinking was:

We've had to do it by ourselves for a while; you know what I mean? It's kind of like sink or swim… when you don't have resources, you still do the work. It doesn't depend on whether I have fat funding or not. We're going to find a way to get lifesaving services to people. We'll just use private foundations to purchase the materials, and then people that were working at the agency kind of had to volunteer their time to get those out to people . . . There was just no staffing money. So we used volunteers quite a bit. We still do. – CBO PT10

### Specific Service Impacts: HIV Testing and Harm Reduction Services

We observed marked differences in the degree to which certain services were or were not transitioned to telehealth delivery. As noted earlier, medical care was generally one domain of service delivery that successfully made the switch. Among our interviewees, harm reduction services constituted one area of operation that often continued with little to no interruption. There was implicit consensus that people who inject drugs would need continued access to sterile syringes and other injection equipment, especially anti-overdose medication, regardless of the worldwide public health crisis. Indeed, harm reduction staff were considered essential workers which allowed them to be “in the field during the entire pandemic.” One individual involved in mobile prevention services would not even consider suspending services: “Nope. I’m going out tomorrow. I’m packing up my car and doing this.” Participants indicated that they were aware of other syringe exchange providers that were forced to close. Our interviewees mentioned an influx of new clients to their facilities as a consequence of the closures.

Service providers invented creative ways to offer harm reduction supplies. One organization created a mail-order delivery system for them. COVID-19 was the catalyst for another agency to switch to mobile syringe exchange. The pandemic effectively served as the helpful push the organization needed to offer services in a format they had hoped to implement anyway. Other organizations shifted from in-person, indoor exchange, to outdoor service delivery ie, curbside or front porch exchange to comply with social distancing requirements. These creative adaptations were not always fully successful and may have thwarted access for some clients:

People now have to call on the phone, even if they're standing out in the front yard, make their order, and then have the transfer of materials and information on the front porch. So it's not an ideal situation, especially if there's a line of people who need services. – CBO PTI0

Another informant elaborated on the challenges that clients encountered with curbside services:

The curbside pickup has made people kind of uncomfortable at times because they feel like coming inside the building might be a more comfortable option when receiving these kinds of supplies. And we know, we provide people with a paper bag full of supplies, or another sort of bag that doesn't really show what they're getting. But there has been this kind of feeling of stigma or shame walking up and getting these curbside services and not being inside the building where they have this sense of privacy. – CBO PT02

To address these challenges, organizations described changing their dispensing protocols to reduce the frequency in which clients had to visit the syringe exchange program, or in the case of challenges specific to mobile service delivery, to reduce the frequency and distance that staff had to travel.

While syringe exchange services continued to be utilized at a high level, interviewees reported far fewer individuals calling about or presenting for HIV testing services. Additionally, it was not as easy to adapt in-person HIV testing protocols (to be either physically distanced onsite or home-delivery based) as it was it was for traditional telehealth encounters or syringe exchange. Even though home or self-HIV testing kits were available prior to COVID-19, individuals were not accustomed to self-administered testing. This needed to be taken into account and resources needed to be developed to provide clear instruction (and encouragement) on how to operate a home-based HIV test kit. Both clinics and CBOs also needed to find ways to advertise access to HIV self-test kits, develop protocols to ship kits to patients/clients, and to repurpose funds to support these changes. None of these efforts were easily implemented regardless of the type of institution.

We got a lot of guidance from the State initially about the syringe exchange program, so I felt pretty comfortable navigating that, but the testing piece was a big unknown. Then we heard there were these in-home testing happening, so our prevention director reached out to the State. – CBO PT07-08

In one case, a fully volunteer-run, high-volume HIV and STI testing site stopped offering testing and treatment services altogether because they felt they did not have the right expertise in COVID-19 infection control and did not want to risk exposing their clients or volunteers should they remain open. This same organization ultimately upended their traditional services and instead began engaging in what they called “mutual aid disaster relief,” distributing food, tents, hygiene kits and setting up handwashing stations.

### Influential Forces Underlying COVID- 19 Impact on Service Delivery

#### Funding: Influx of Federal, State, and Local Financial Support Facilitated Adaptation

The clinics represented in our study adopted new processes and workflows to ensure compliance with COVID-19 safety guidelines and to remain operational. Quickly adapting to the reality of public health measures was possible, in part, because of the influx of financial support from federal, state, and local sources, including private foundations. In many cases, the COVID-19 funds provided through the Coronavirus Aid, Relief, and Economic Security Act (CARES Act) were dispensed with modest spending restrictions.^11^ As participants described, historically, funders would develop contracts that dictated spending priorities and determined what constituted eligible expenses. With the onset of the pandemic, clinic leadership were given much more autonomy to decide how to spend additional funds, as well as to repurpose existing funds. They were entrusted to determine the priorities based on the needs of the local context.

Funds were most immediately spent on purchasing personal protective equipment and hygiene supplies. Clinics also spent money retrofitting clinic spaces to be able to offer at least some amount of in-person services including the purchasing of air purification systems and thermal checking devices. Aside from these COVID-19 specific expenses, clinics made decisions according to their unique organizational and community needs. Some examples included spending funds to retain rather than furlough staff, offering staff bonuses, renting additional building space to offer in-person services in a socially distanced manner, and purchasing phones for patients to enable them to connect to medical and supportive services.

One interviewee described the flexible stance adopted by their local health department -- “the county changed what hitting a goal means” in response to the new challenges that patients faced when accessing care during the pandemic. For example, one change allowed patient visits to occur every six, rather than every three, months for patients to be considered engaged in care. Laboratory-only visits became allowable. Purchasing certain over-the-counter medications for patients became allowable. These flexibilities were described as translating into tangible evidence of patient-centered care, as highlighted by a key informant: “That sounds like a little thing. But it's really been a big thing. I think the patients feel much more supported than they did before*.*”

Clinics prioritized resources in distinct ways when considering how to make the best use of newly available funds. Developing a list of priorities was not difficult. Before the COVID-19 crisis, representatives from each of these clinics were keenly aware of the significant unmet needs of a certain proportion of their patients. They attempted to assist in meeting these basic needs. However, with the advent of COVID, the extent of the unmet needs broadened – more patients struggled to meet basic needs and faced additional stressors. Flexibility in the use of funds allowed representatives from these clinical settings to attempt to meaningfully meet and fulfill these needs in a much more significant way than was possible under typical funding environments.

Some of the RW-funded CBO representatives also spoke about the critical importance of new monies offered with few restrictions as well as increased flexibility with the use of existing funds. This perspective was mostly limited to the CBOs that were Ryan White grant recipients and administered housing grants through HOPWA or provided non-medical case management services. For example, we heard about the benefits of loosened regulations for HOPWA funds (eg, allowing inspections to happen remotely) as well as a general appreciation that they were given the autonomy to spend money to benefit clients in whatever way they deemed necessary. Below, RW-funded CBO-based informants spoke about the benefits of greater flexibility:

We're lucky because HOPWA made adaptations at the beginning of COVID where they allow certain things to happen in a way they wouldn't normally do it. It's a federal government agency, and they're very strict, but they were able to see that the need was great and make those adaptations where it helps us as we approach our clients…. And we were really lucky too because in the beginning, well and just generally, a lot of the grants and funding opportunities became really straightforward, and they weren't asking questions. It was just like, “Use it how you need to spend it.” – CBO PT07-08

The addition of the COVID money has been so helpful. I can't emphasize that enough. Mainly because all of the agencies, for the first time that I've ever seen with the federal government, decided to drop their normal protocols, you know, loosen up the rules . . . Do what you have to do. Just help people. A very different way of looking at things . . . They're taking all the regulations away and allowing us to pay for what we had to pay for. – CBO PT11

Because these additional funds were offered with fewer restrictions or rules, RW-funded CBOs were able to set aside worries about the viability of their organizations. As one informant shared about their organization, “we weren’t freaking out about our own sustainability” which allowed them to prioritize meeting the needs of their clients as well as providing resources for their staff. Similar to clinic key informants, RW-funded CBO staff reported purchasing laptops, phones, and tablets for staff to be able to continue to provide services.

Then we got some COVID money through the Ryan White Care Act, and I was able to buy iPhones and iPads for my staff so that they could do a better job of keeping in touch with their clients on a one-on-one situation. And that's gone really well; I was really surprised. – CBO PT11

Furthermore, by offering patients and clients phones, staff were able to send friendly messages to patients as a form of connection eg “Happy Monday – hope you have a great week.” And it gave patients the ability to interact with clinic staff such as patient navigators or outreach workers when they would otherwise have little to no means of doing so without having been given a phone by the clinic.

#### Relationships: Leveraging Existing Networks Mitigated Impact on Services

Participants described the importance of relationships with other clinics, agencies, and community organizations in adjusting to a changing landscape and meeting the needs of clients. These creative partnerships were especially critical to CBOs that often had limited financial resources. One participant described how their friendships with other organizations and a willingness to provide in-kind services in return helped them maintain community events during shelter-in-place:

I don't have any money as an organization. But you know what we do have? We have friends. So, we reach out to our friends. Like we reached out to the [local arts center] so that we could use their parking lot to do a movie for the LGBTQ community so that everybody could do a drive-in in their parking lot. We didn't have a big enough parking lot. We didn't even have money. They had money. They had a big screen. They said, "yeah, you can use it. Just partner with us on this document." …You have to have an understanding of the inter-agencies in order to put these things into play. – CBO PT09

Another CBO-based informant described how connections to other organizations and case managers in the area had helped them connect clients to scarce resources:

I'm working with the family right now that was literally on the street. I got a call from a case manager at one of our local hospitals. So, I worked with the hospital, getting to know the case managers, and if they have someone who has a need outside of health care for HIV, they give me a call in this area. And so, that particular [family], I was able to sustain, get them out of their homeless situation, get them into a hotel or motel, work with another agency that has more money than I do, and so we're, like, working in collaboration. – CBO PT13

Like CBOs, clinics relied on their existing networks to meet the needs of their patients; in some cases, they built new partnerships. For example, one clinic described how when a nearby gym closed during COVID, they were able to rent the unoccupied space, which afforded them more room and allowed substance use support groups to continue to meet in-person while maintaining physical distancing guidelines. Other agencies harnessed needed essential services through mobilizing their advocacy networks in order to change existing policy, broadening access for their clients. In this case, food insecurity was a widespread need identified during COVID, and service providers were able to lobby to make meals accessible regardless of disability status during the pandemic.

So, we referred clients to home-delivered meals. We have a partner agency that gets Ryan White funding. And if we had a client that was determined by their medical provider that they were not able to cook for themselves, then the doctor would sign off an application, get that program started for the client through their case manager. That has shifted with COVID. Now they made it more flexible through advocacy through the [local] HIV planning group… We saw more clients being mentally and emotionally unable to do that level of meal preparation. So, they allowed for anyone that needed that home-delivered meal to be an option, regardless of their physical disability, for example. – Clinic PT16

#### Workforce Vulnerabilities: Turnover, Burnout, Compounding Stressors

We observed significant variation in the impact of COVID-19 on the HIV workforce. Whenever possible, organizations and departments committed to maintaining staff at existing levels rather than resorting to furloughs. These actions were either led by or strongly supported by the organizations’ boards. Many managed to maintain their staff. For example, one clinic used some of its federal aid dollars to provide each employee with a $2000 bonus. Another clinic’s board offered staff more time off. A third clinic headed off furloughs for their HIV staff members through strong advocacy within the organization and by suggesting that the HIV staff could transfer their skills in infectious disease to serve as COVID-19 health educators.

It's been challenging on a lot of levels, but it's also been very rewarding for everybody involved. My staff works as a team. We talk every day… I think that level of mutual support and understanding has made a real difference in their ability to keep the right attitude and to provide the best services they can for their clients. – CBO PT11

Some providers or staff did leave their positions due to various reasons, eg, having to care for other family members, or work changing to a format that was unappealing. Importantly, there were compounding stressors most typically experienced by individuals from historically marginalized communities. These individuals appeared to be disproportionately impacted by COVID. This was the case for service providers who were living with HIV. The desire to be physically present to provide services to clients was complicated by concerns about their own health and wellbeing as described by the following participant:

Many of us gay men that work here - not all, but many - we are HIV positive. So, all you needed to hear was … "compromised immune system," and that ran like fire here. We felt like we were just walking sponges of COVID, you know? And so, to answer your question, it was tough to deliver a service to an anxious community when the providers were anxious themselves. That was just hard to do. – Clinic PT14

In nearly all interviews, we observed an unwavering commitment to compassionately serve individuals living with HIV and people who use drugs. For example, retention specialists were actively working to locate patients and were not concerned about their risk of exposing themselves to COVID. Instead, they wanted to serve their patients. This was particularly heightened in rural communities where we observed a tight knit community among CBO employees who also forged close relationships with their clients.

## Discussion

The clinics represented in this study demonstrated an ability to pivot quickly and effectively when shelter-in-place orders went into effect in the spring of 2020. The CBOs, by contrast, had less infrastructure and funding to accommodate a rapid shift to virtual service delivery. In some cases, virtual service delivery was undesirable or simply not feasible to meet clients’ needs in a virtual environment. The ability of CBOs and HIV-related clinics to provide continuity of services for clients/patients was due in large part to: 1) the infusion of additional federal, state and/or county funding and 2) the lifting of restrictions on how to spend federal, state and county funds. Ryan White funded organizations benefited from the supplemental monies offered through the US Congress CARES Act. Interview participants frequently emphasized that organizations benefited from the flexibility and attendant trust that funders afforded them to make determinations about how best to allocate resources to meet the needs of their clients. Our study participants representing organizations without a Ryan White program designation re-configured their service delivery models as best they could by relying on social networks, dedicated volunteers, and paid staff. With the exception of harm reduction services, they were not able to maintain pre-COVID levels of service.

The challenges with specific services exemplified the way that the pandemic intersected with agency capacity and existing client vulnerabilities. For example, curbside delivery of harm reduction services offered a potential strategy for continuing syringe exchange but placing the service outside left some clients feeling vulnerable to stigma. Elsewhere, demand for HIV testing declined heavily. The challenges with testing bear some consideration as it historically has been a service offered on a drop-in basis to facilitate delivery to clients from vulnerable populations. With the push to routinely screen all individuals ages 13–64, HIV testing is also now available in a variety of settings from CBOs to sexual and reproductive health clinics to emergency departments. Although the technology to self-test is available, it is not in widespread use. It, thus, is not surprising that facilities experienced a reduction in the rates of HIV testing during the shelter-in-place. In fact, in the initial quantitative survey of California agencies, which preceded this study and from which interview participants were drawn, 57% reported a reduction in HIV testing and 34% in STI testing.^12^ This mirrors the limited data in the literature on the impact of COVID on HIV testing rates in the United States.^13, 14^ One study across four US states found that HIV testing declined by 68–97% when shelter-in-place went into effect, and continued to remain lower than pre-pandemic levels (11-54% reduction in testing) as shelter-in-place orders were lifted.^13^ Our qualitative findings provide us with a glimpse into the attempts that clinics and CBOs made to restore access to testing. Only one agency completely discontinued their testing services for a period of time. The others made efforts either to create safe strategies for in-person testing or to purchase and distribute self-testing kits. However, in spite of these efforts to establish or re-establish access to HIV testing, challenges related to uptake persisted. It remains to be seen whether clients found HIV self-test kits to be valuable. More research on understanding attitudes about HIV self-testing during COVID among individuals with a history of place-based HIV testing is needed.

Other adaptations made by clinics on behalf of their patient population living with HIV appeared to have generated *greater* forms of patient-centered care by removing known barriers to engagement in HIV care that existed prior to COVID. Some of these notable changes reported here and in a Kaiser Family Health Foundation Report^15^ include allowing Ryan White recertifications to happen over the phone, expanding flexibility for telehealth services, greater flexibility with HOPWA, allowing multi-month prescription refills for ART and PrEP, expanding at home HIV testing, and reducing viral load testing frequency. Changes such as those made to the housing re-certification process allowed patients to maintain their housing and thereby was perceived to facilitate retention in HIV care. Removing the barriers to maintaining one’s eligibility by allowing different methods of verification that were favorable to patients rather than onerous led to *stability rather than uncertainty*.

Because clinics were allowed to use funds to purchase cell phones for patients who were experiencing homelessness, a formerly unallowable expense, clinic and CBO staff could maintain communication with patients and clients they considered to be the most vulnerable and at risk for poor health outcomes during COVID-19. These individuals may have been otherwise out of reach and unable to attend telehealth visits.

Our qualitative findings align with the results of a Kaiser Family Foundation survey conducted among Ryan White providers.^15^ They found 99% of providers reported an “immediate pivot” to providing telehealth services and attributed the influx of Ryan White CARES Act funding as “critical” to their ability to quickly adapting to this new mode of care delivery despite having little to no prior experience with telehealth. They also reported on the difficulties some providers had learning to use the technology as well as the challenges of maintaining staff morale. The findings herein tell the story of the specific impacts COVID-19 had on California HIV care and service providers and their responses to it, including what it was like for community-based organizations to determine whether and how to reconfigure their services to a virtual setting. Our findings reflect similar challenges outlined in research conducted on the use of telehealth in HIV care settings, such as technological challenges, low levels of digital literacy or absence of technology among service providers and patients.^16^ In addition, our findings echo results reported on the loosening of regulatory and payor agencies restrictions as a facilitator of rapid expansion of telehealth^17^ as well as those of the influential Infectious Disease Society of America who in 2021 strongly encouraged the continued use of telemedicine for HIV because these visit types could lead to a dramatic reduction in barriers to HIV care.^18^

The literature on the impact of COVID-19 on CBOs is sparse, however, our findings align with some observations made by Pinto and Park in 2020, for example, CBOs faced resource shortages, low staff morale and communication challenges with clients due to lack of access to high-speed internet or cellular phone plans.^19^

### Implications

There are several implications from this study that apply to health care delivery and policy settings in a post-pandemic world. These data clearly suggest that an enhanced level of flexibility within funding streams and reporting requirements should be continued. Similarly, authorizing 90-day prescription refills, and full reimbursement for telehealth appointments actually led to better care engagement and treatment adherence for some previously difficult to engage patients. Recreating services to make them more client-centered and flexible may be of value to healthcare authorities charged with dispensing as well as withholding resources to patients because they lead to better HIV-related health outcomes. Furthermore, maintaining some of the flexibility that was necessary to retain patients in care during COVID may also help to break down medical mistrust issues and disdain for institutions that have created harm in the past. Indeed, this may be an opportunity to redress some of the harms inflicted knowingly or unknowingly by organizations, medical or otherwise upon vulnerable populations.

The changes we recommend maintaining in order to continue providing maximally patient-centered care as well as in preparation for a future pandemic include ensuring that clinics have technology in place to conduct telehealth visits and a workforce trained to make optimal use of telehealth. Simultaneously, payer sources must allow for billing of such services. We also recommend that funders institute allowances for greater ownership over how a portion of funds are spent so that clinics and CBOs can tailor resources to the local needs of patients/clients. Finally, we recommend that clinic or CBO managers routinize burnout prevention strategies such as flexible work schedules, hazard pay, provision of childcare and easy access to behavioral health resources.

### Limitations

Our sample did not include interviews with people living with HIV and though some key informants discussed the hardships faced in trying to reach patients, we do not have first-hand accounts of what it was like to be on the receiving end of these services.

## Conclusions

COVID-19 forced health care leaders to think strategically about how to best serve clients under extraordinary circumstances. Our findings suggest that HIV-affiliated organizations and their patient/client population benefited from a more flexible approach to HIV-related care. Those organizations and patients who adapted to telehealth have expressed a desire to have ongoing access to telehealth services. On the other hand, some organizations were pushed to the limit in terms of funding and capacity. Building relationships with local agencies was critical for some organizations during the early days of the pandemic. Further nurturing and extension of these partnerships may help resource-constrained organizations and ensure better coordination of care and services for clients. These recommendations extend beyond the acute phase of the COVID response, rather they should be kept in place for the long run benefitting these organizations and the populations they serve well into the future.
